# Indole-3-acetic acid enhances ruminal microbiota for aflatoxin B1 removal *in vitro* fermentation

**DOI:** 10.3389/fvets.2024.1450241

**Published:** 2024-12-20

**Authors:** Jiajin Sun, Zhonghao Wang, Xinyu Yan, Yuqi Zhao, Li Tan, Xuning Miao, Rong Zhao, Wenjie Huo, Lei Chen, Qinghong Li, Qiang Liu, Cong Wang, Gang Guo

**Affiliations:** College of Animal Science, Shanxi Agricultural University, Taigu, China

**Keywords:** indole-3-acetic acid, aflatoxin B1, rumen fermentation, rumen microbiota, removal rate

## Abstract

Aflatoxin B1 (AFB1) has been recognized as a serious health risk for ruminant animals. From a molecular perspective, indole-3-acid (IAA) possesses the potential to enhance the removal of AFB1 by rumen microbiota. Therefore, this study aims to investigate the impact of different concentrations of IAA on the removal of AFB1 by rumen microbiota using an *in vitro* technique. Experiment 1: interaction between AFB1 and rumen fermentation. Experiment 2: The study used a randomized design with five IAA levels (0, 15, 150, 1,500, and 7,500 mg/kg) to examine the effect of IAA on AFB1 removal and its impact on rumen fermentation. The results showed: (1) the content of AFB1 gradually decreased, removal rate of up to 75.73% after 24 h. AFB1 exposure altered the rumen fermentation pattern, with significantly decreased in the acetic acid/propionic acid ratio (*p* < 0.05). It significantly reduced the relative proportions of *R. amylophilus*, *P. ruminicola*, and *F. succinogenes* (*p* < 0.05). (2) As the content of IAA increased, AFB1 exposure decreased. A total of 15 and 150 mg/kg IAA significantly mitigated the negative impact of AFB1 on key rumen bacteria (*R. amylophilus, P. ruminicola* and *F. succinogenes*), increased acetate levels and acetate/propionate ratio (*p* < 0.05). However, 1,500 mg/kg IAA lowered levels of propionate and isovalerate, adversely affected enzyme activities (pectinase, xylan and Carboxymethyl-cellulase) and relative proportions of microbiota (*R. flavefaciens*, *P. ruminicola* and *F. succinogenes*). In conclusion, IAA significantly removed AFB1, and in the range of 150 mg/kg of IAA reduced the negative effects of AFB1 on *in vitro* fermentation characteristics and fermentation end-products.

## Introduction

Aflatoxin B1 (AFB1) are toxic secondary metabolites produced during the growth of molds and commonly found in grains and animal feed (1–3). AFB1 exposure may reduce feed intake and weight gain, disrupt rumen microbiota balance, impair liver and kidney function, and negatively affect overall health (4, 5). Additionally, AFB1 can be transferred into animal products (meat and milk), thereby posing a risk to human health (6, 7). A recent review by Eskola et al. (8) suggested that about 60 to 80% of the global food crops are contaminated with mycotoxins. In the subtropical region, the highest concentration was 3.76 mg/kg (9). Ma et al. (10) collected 742 feed ingredients samples from various regions of China. Among them, more than 83.3% of the samples was contaminated AFB1 at different concentrations. The highest concentration in China exceeded the standard of 1.6 mg/kg (9). Previous studies had shown that rumen microbiota have the ability to remove AFB1 (7, 11, 12), but the ability is limited. Therefore effective methods of enhancing the removal capacity of the original rumen microbiota for AFB1 are necessary to be developed.

Regarding the food safety, dietary interventions with plant-derived additives are a promising approach to promoting the removal of AFB1 by rumen microbiota. In mammals, Indoleacetic-3-acid (IAA) is an important indole-derivative catabolized from dietary tryptophan by the intestinal microbiota, but intestinal dysbiosis can influence IAA production (13). IAA has various special functions in microbiota metabolism (14, 15), and has a certain regulatory effect on the synthesis of tryptophan and cytochromes (16). Cytochrome CYP can oxidize AFB1, forming AFB1-8, 9-epoxide (17, 18). Moreover, IAA was able to restore the intestinal microbiota balance and maintain its stability (19). But there are few reports on the removal of rumen microbiota and AFB1 by IAA. The objective of the present study was therefore to evaluate different dose of IAA in the rumen effect of the removal rate, ruminal fermentation profile, enzyme activity, and microbiota *in vitro*.

## Materials and methods

### Experimental design, procedure, and sampling

Experiment 1: They were divided into 80 bottles and allocated to two groups: (1) the CK group, blank control group without AFB1; (2) the AFB1 group, with 1 mg/kg AFB1. The *in vitro* fermentation was independently conducted seven times for 0, 1, 2, 3, 4, 5, 24, and 48 h. Experiment 2: The removal rate of AFB1 in Experiment 1 was used to select 24 h as the fermentation time for Experiment 2. They were divided into 20 bottles and allocated to five groups: (1) the AFB1 group, with 1 mg/kg AFB1; (2) 1 mg/kg AFB1 + 15 mg/kg IAA group; (3) 1 mg/kg AFB1 + 150 mg/kg IAA group; (4) 1 mg/kg AFB1 + 1,500 mg/kg IAA group; (5) 1 mg/kg AFB1 + 7,500 mg/kg IAA group. Each treatment was performed inquintuplicate. The fermentation liquid was collected and stored in liquid nitrogen, pre-column derivatization (20) was carried out to detect AFB1 content and rumen fermentation indicators.

*In vitro* ruminal fermentation followed the method described by Longland et al. (21). The rumen fluid was collected from three Holstein dry cows (650 kg ± 20 kg) before the morning feeding. The ingredients and chemical composition of total mixed ration are presented in Table 1. The mixture of the rumen fluid was filtered by four layers of gauze, mixed with buffer solution (v/v = 1:1), and kept at 39°C in a water bath while continually flushed with CO_2_. A total of 0.2 g alfalfa silage (DM, 364.20 g·kg^−1^ FM; CP, 173.2 g·kg^−1^ DM; NDF, 389.5 g·kg^−1^ DM; ADF, 285.5 g·kg^−1^ DM) and 0.2 g starch (DM, 85.24 g·kg^−1^ FM; Starch, 612.1 g·kg^−1^ DM; CP, 55.8 g·kg^−1^ DM; NDF, 87 g·kg^−1^ DM; ADF, 21.9 g·kg^−1^ DM) as the fermentation substrate and placed in nylon bags. Afterwards, 60 mL of the diluted rumen fluid was divided into individual fermentation bottles, sealed, and placed in a shaker (120 rpm) at 39°C (22). Upon at 24 h, it was immediately removed, stopped in an ice bath, and samples were taken for detection of various indicators.

**Table 1:** Ingredients and chemical composition of a total mixed ration.

Items	Total mixed ration (TMR)
Ingredients (g kg^−1^ DM)	
Oathay	213.5
Chinesewildrye	178.4
Wholeplantcornsilage	458
Soybeanmeal	30.5
DDGS	25.5
Wheatbran	50.9
Vitamins and minerals^1^	43.2
Chemical composition^2^	
DM (g kg^−1^ FW)	462.2
CP (g kg^−1^ DM)	114
NDF (g kg^−1^ DM)	460.5
ADF (g kg^−1^ DM)	303.5
Ca (g kg^−1^ DM)	4.2
P (g kg^−1^ DM)	2.3

^1Contained VA10000IU, VD2000IU, VE 50 mg, Fe 90 mg, Cu 12.5 mg, Mn 30 mg, Zn 90 mg, Se 0.3 mg, I 0.5 mg, Co 0.3 mg of vitamin E per kg TMR. 2DM, dry matter; FW, fresh weight; OM, organic matter; CP, crude protein; NDF, neutral detergent fiber; ADF, acid detergent fiber. The values of chemical composition are means of triplicate determinations.^

### Fermentation indicators, AFB1, fiber-degrading enzyme activity, and qPCR

A total 1 mL of the collected rumen fluid was subjected to pre-column derivatization (20). The content of AFB1 was determined using an Agilent 1,260 Infinity II high-performance liquid chromatograph (HPLC) with a C18 column, 4.6 × 250 nm, 5 μm. The mobile phase was acetonitrile-water (20:80); column temperature: 40°C; mobile phase flow rate: 1.0 mL/min; injection volume: 20 μL; excitation wavelength 360 nm, emission wavelength 440 nm; detection time 20 min. The degradation rate of AFB1 = (AFB1 content in the blank control - AFB1 content in the experimental group)/AFB1 content in the blank control × 100%.

pH was measured using a pH meter (LE438, Mettler Toledo Instruments Co., Ltd. China). For the other analyses, 25% meta-phosphoric acid was added to the fermentation fluid (1/5, *v*/*v*), and then samples were centrifuged for 10 min at 10,000× *g* at 4°C using a high-speed freezing centrifuge (Eppendorf 5810R, Eppendorf AG, Hamburg, Germany). The supernatant was collected and stored at −80°C for NH_3_-N and VFA determinations. The content of ammonia nitrogen (NH_3_-N) was determined using the phenol-hypochlorous acid colorimetric method (23). The content of volatile fatty acids (VFA) (Acetic acid, Propionic acid, Butyric acid, Isobutyric acid, Valeric acid and Isovaleric acid) was determined using a high-performance gas chromatograph (GC-TRACE 1300, column model 30 m × 0.25 mm × 0.25 μm) (24). The determination of the four types of cellulase (Carboxymethyl cellulase, Pectinase, Xylanase, *α*-glicosidase) was carried out according to the method described by Agarwal et al. (25).

Ruminal microbial DNA was extracted according to the method of Edrington TS (26). qPCR (primers were listed in Table 2) was used to determine the relative abundance of 10 bacteriain the incubation fluid. Real-time PCR was carried out on an Applied Biosystems Step One Plus Fast Real-Time PCR System (Applied Biosystems Co., USA). The reaction mixture (20 μL) were mixed with SYBR Premix *Taq*TM (10 μL, TaKaRa Biotechnology Co., Ltd., Dalian, China), ddH_2_O (7.0 μL), PCR forward or reverse primer (0.2 μmol L^−1^, 0.8 μL), ROX Reference Dye (0.4 μL, 50×) and the template DNA (1 μL). The number of cycles required to reach a threshold adjusted for each taxon (Ct) was recorded for eachsample. The PCR programs included initial denaturation (1 cycle of 50°C for 2 min and 95°C for 2 min), primer annealing and product elongation [40 cycles of 95°C for 15 s and 60°C for 1 min (27, 28)].

**Table 2:** PCR primers for real-time PCR assay.

Target species	Primer sequence (5′)	GeneBank accession no.	TE (°C)	Size (bp)
Total bacteria	F: CGGCAACGAGCGCAACCC R: CCATTGTAGCACGTGTGTAGCC	AY548787.1	60	147
Total anaerobic fungi	F: GAGGAAGTAAAAGTCGTAACAAGGTTTC R: CAAATTCACAAAGGGTAGGATGATT	GQ355327.1	57.5	120
Total protozoa	F: GCTTTCGWTGGTAGTGTATT R: CTTGCCCTCYAATCGTWCT	HM212038.1	59	234
Total methanogens	F: TTCGGTGGATCDCARAGRGC R: GBARGTCGWAWCCGTAGAATCC	GQ339873.1	60	160
*R. albus*	F: CCCTAAAAGCAGTCTTAGTTCG R: CCTCCTTGCGGTTAGAACA	CP002403.1	60	176
*R. flavefaciens*	F: ATTGTCCCAGTTCAGATTGC R: GGCGTCCTCATTGCTGTTAG	AB849343.1	60	173
*B. fibrisolvens*	F: ACCGCATAAGCGCACGGA R: CGGGTCCATCTTGTACCGATAAAT	HQ404372.1	61	65
*F.succinogenes*	F: GTTCGGAATTACTGGGCGTAAA R: CGCCTGCCCCTGAACTATC	AB275512.1	61	121
*R. amylophilus*	F: CTGGGGAGCTGCCTGAATG R: GCATCTGAATGCGACTGGTTG	MH708240.1	60	102
*P. ruminicola*	F: GAAAGTCGGATTAATGCTCTATGTTG R: CATCCTATAGCGGTAAACCTTTGG	LT975683.1	58.5	74

### Statistical analyses

Data of experimental 1 using Statistical Analysis System (paired sample *T*-test). Data of experimental 2 using the general linear model (GLM) procedure of Statistical Analysis System (SAS, 1999). The repeated measures model accounted for the fixed effects at different level of treatment. Tukey’s test was used for the multiple comparisons among mean values and linear and quadratic effects were calculated at *p* < 0.05, (*p < 0.05; **p < 0.001). *p* < 0.05 was accepted as statistically significant, and *p*-values between 0.05 and 0.10 were considered to represent a statistical trend. Figures were drawn using KingDrawPc V3.0.2.20 (Qingdao Qingyuan Precision Agriculture Technology Co., Ltd., Qingdao, China), GraphPad Prism 9.0 (GraphPad Software, San Diego, CA, USA), and Adobe Illustrator 2022–26.0 (Adobe Systems, San Jose, CA, USA) for graphic illustration.

## Results

### Interaction between AFB1 and rumen fermentation

**Figure 1:**
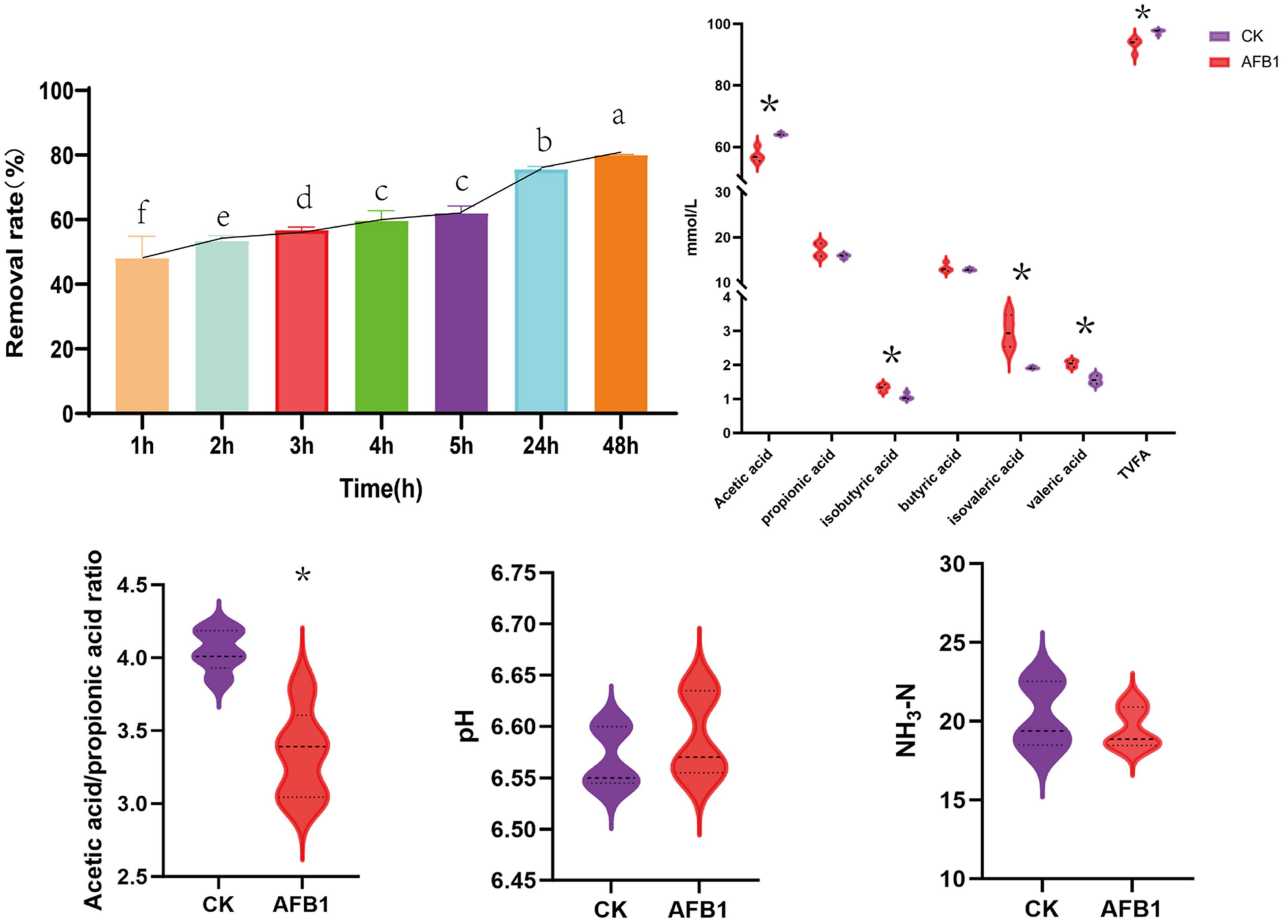
Interaction between AFB1 and rumen fermentation. CK: blank control group without AFB1, AFB1: with 1 mg/kg AFB1. *p < 0.05; **p < 0.001. Different letters indicate a significant difference.

The removal efficiency of AFB1 exhibited a stable and significant increasing trend with the extension of incubation time (Figure 1). The removal rate of AFB1 increased rapidly in the first 24 h, after which the rate of removal slowed down between until 48 h. At 48 h, the removal rate of AFB1 can reach 80.09%. Considering the factors of removal efficiency, this experiment selects 24 h as the final fermentation time. Compared to the control group, AFB1 exposure significantly reduced the content of acetic acid (*p* < 0.05); meanwhile, the content of propionic acid, isobutyric acid valeric acid, and isovaleric acid significantly increased (*p* < 0.05). Further analysis shows that AFB1 exposure led to a significant decrease in the acetic acid/propionic acid ratio (*p* < 0.05). In addition, AFB1 exposure did not significantly affect the pH value and NH_3_-N content of the *in vitro* fermentation fluid.

**Figure 2:**
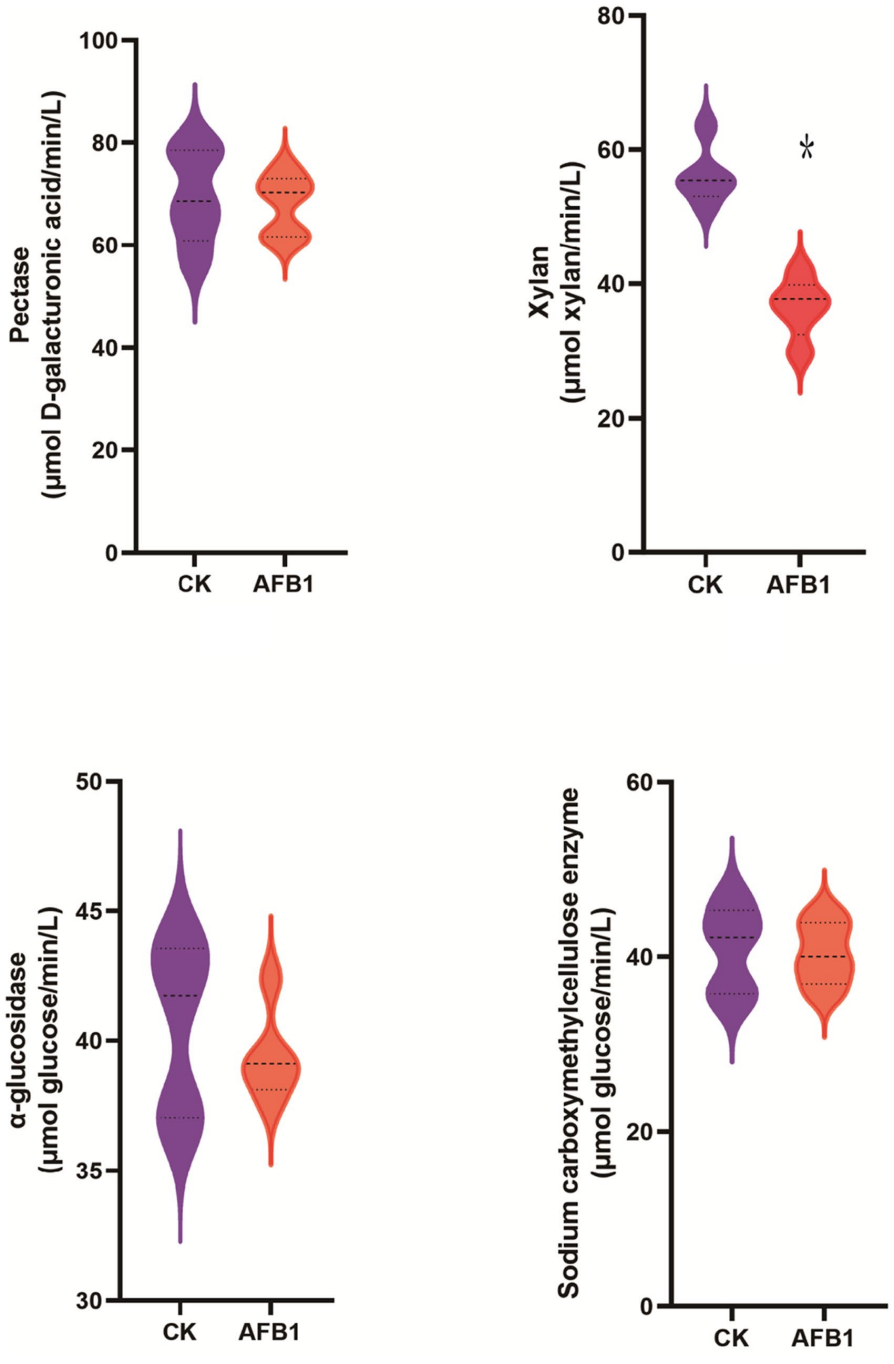
Interaction between AFB1 and rumen fermentation.

Compared to the control group, AFB1 exposure has a significant (*p* < 0.05) effect on reducing the activity of xylanase, while it does not significantly affect the activity of pectinase, carboxymethyl cellulase and *α*-glucosidase (Figure 2).

**Figure 3:**
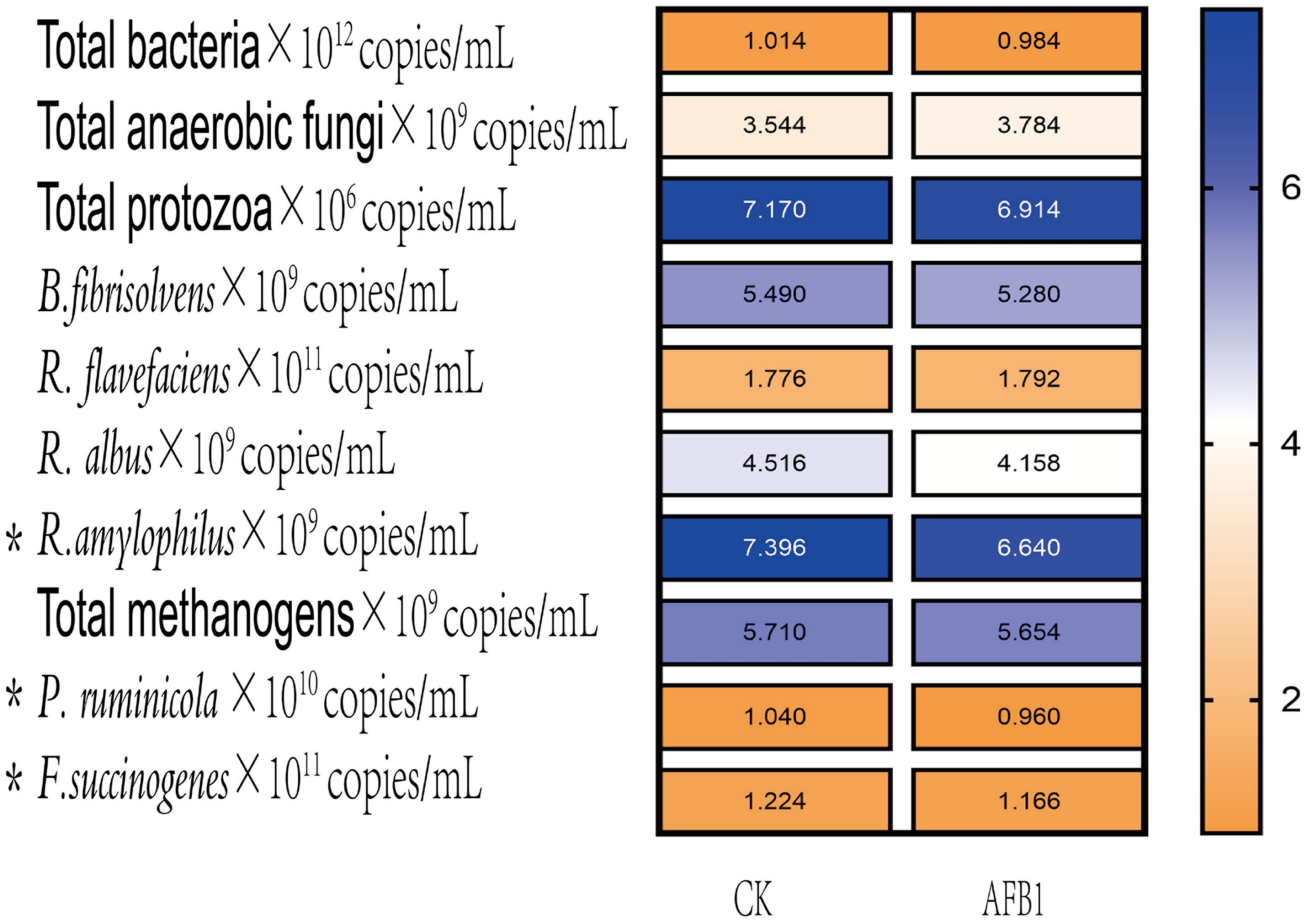
Interaction between AFB1 and rumen fermentation.

As shown in Figure 3, the relative proportions of *Prevotella ruminicola* and *Fusobacterium succinogenes* significantly (*p* < 0.05) decreased after AFB1 exposure, indicating that the presence of AFB1 inhibits the growth of these two types of bacteria.

### Effect of IAA supplementation on rumen fermentation and AFB1 removal

**Figure 4:**
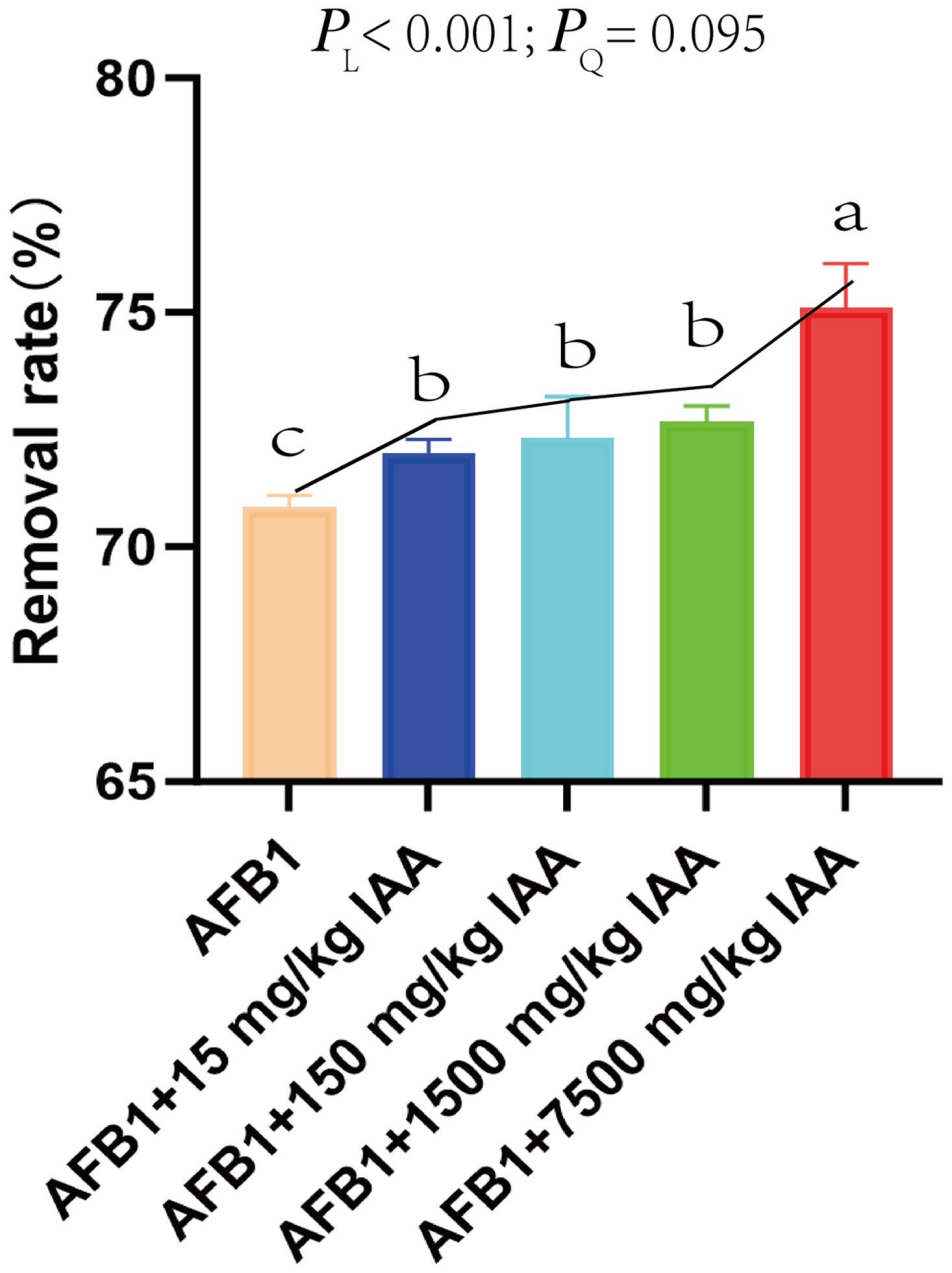
Effect of IAA supplementation on rumen fermentation and AFB1 removal. *P_L_* represents a linear response, *P_Q_* represents a quadratic linear response.

It can be observed that the content of IAA added showed a positive correlation with the removal rate of AFB1 (Figure 4). At concentration of 7,500 mg/kg IAA, AFB1 removal rate reached a maximum of 75.1%.

**Figure 5:**
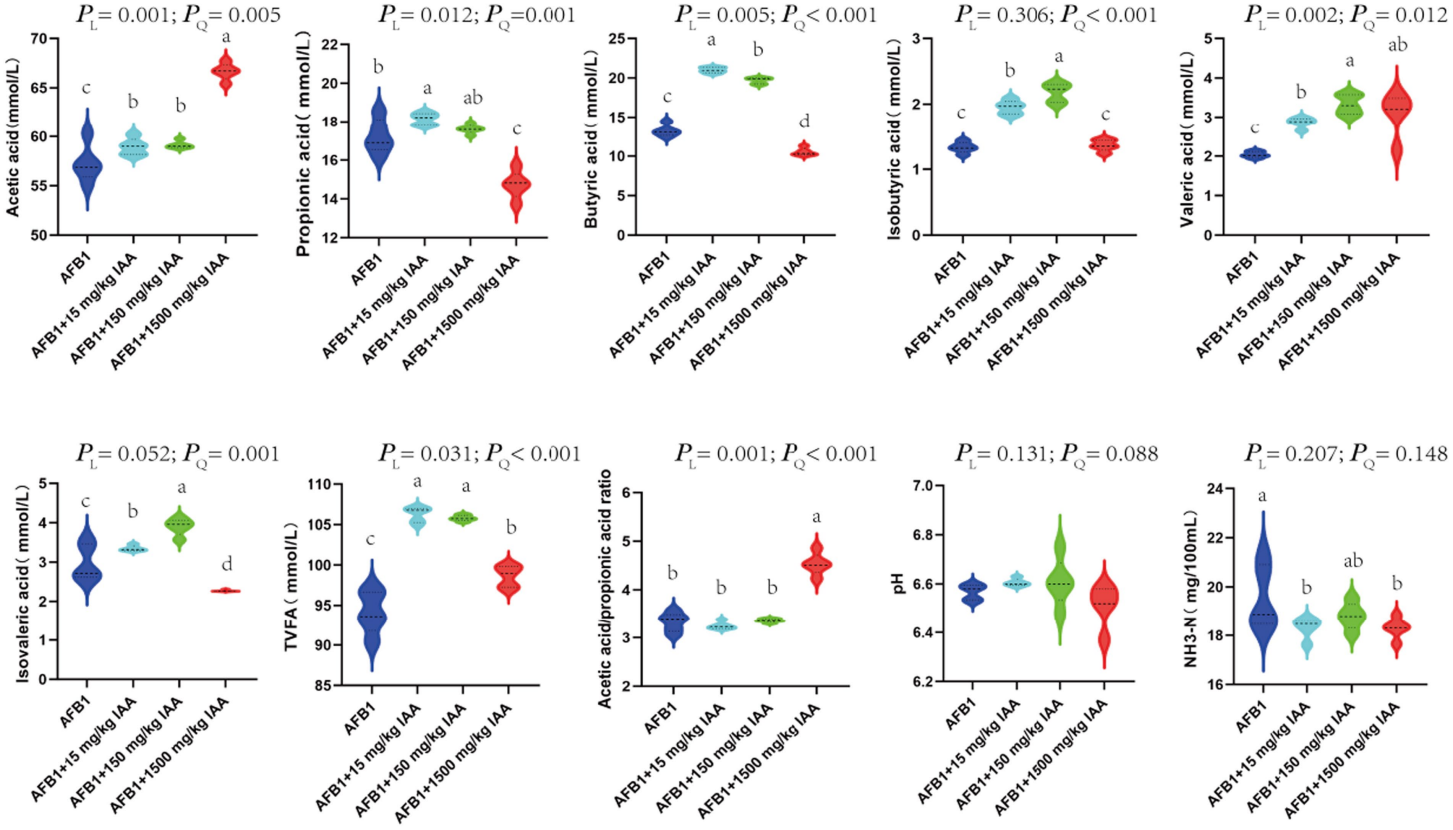
Effect of IAA supplementation on rumen fermentation and AFB1 removal.

IAA at concentrations of 15 mg/kg and 150 mg/kg showed a positive correlation with the content of volatile fatty acids. Specifically, IAA at concentrations of 15 mg/kg and 150 mg/kg had a significant increasing trend in the content of acetic acid (*P_L_* = 0.001), propionic acid (*P_L_* = 0.012), butyric acid (*P_L_* = 0.005), isobutyric acid (*P_Q_* < 0.001), and valeric adid (*P_L_* = 0.002), isovaleric acid (*P_Q_* = 0.001) and total volatile fatty acids (TVFA) (*P_L_* = 0.031). Furthermore, at 1500 mg/kg IAA, the content of acetic acid (*P_L_* = 0.001), valeric acid (*P_L_* = 0.002), the ratio of acetic acid/propionic acid (*P_L_* = 0.001), and TVFA (*P_L_* = 0.031) in the fermentation broth significantly increased, but the content of propionic acid (*P_L_* = 0.012), butyric acid (*P_L_* = 0.005), isobutyric acid (*P_Q_* < 0.001), and isovaleric acid (*P_Q_* = 0.001) significantly decreased trend. The addition of IAA did not significantly affect the pH and NH_3_-N content in the fermentation fluid (Figure 5).

**Figure 6:**
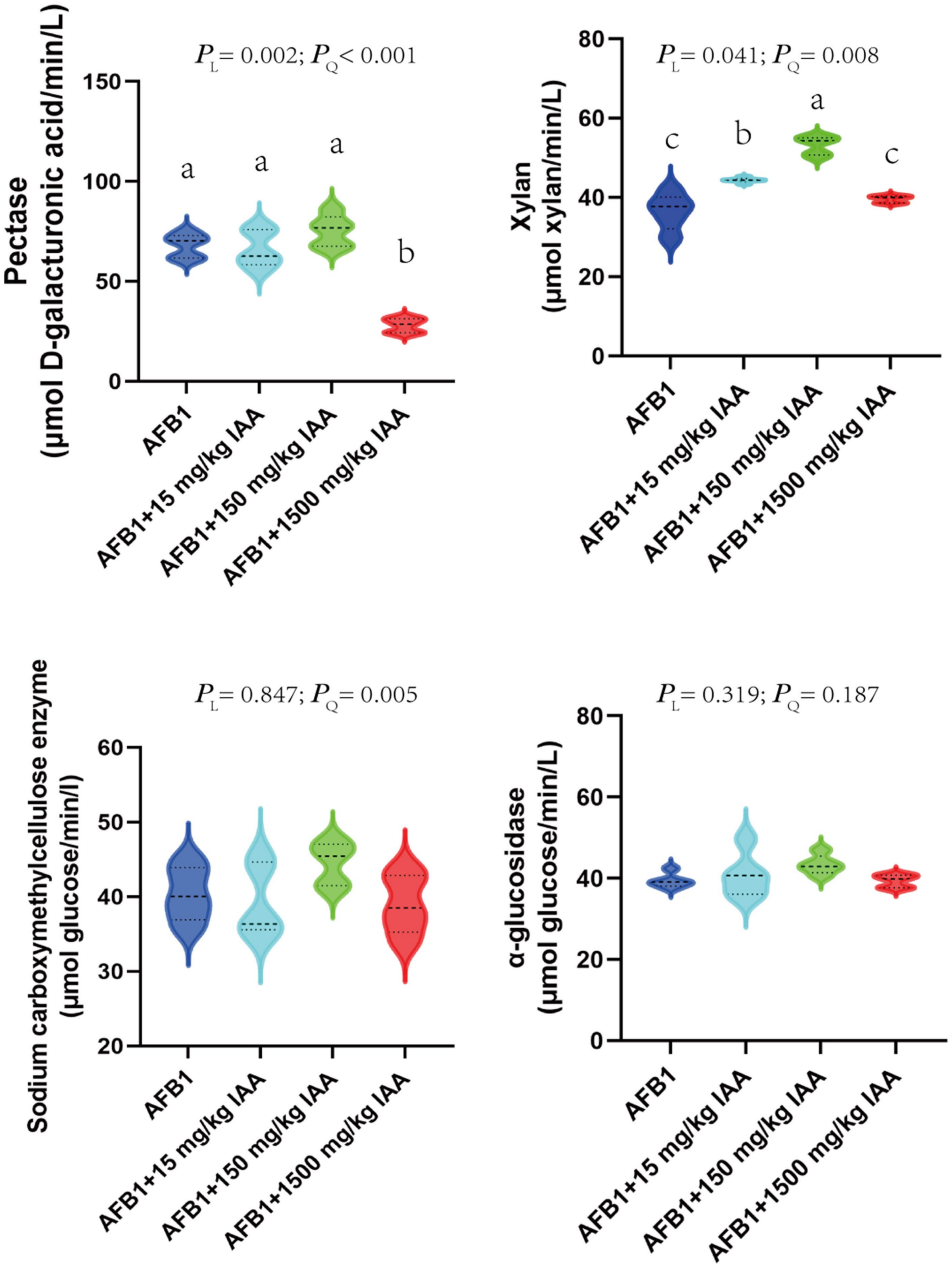
Effect of IAA supplementation on rumen fermentation and AFB1 removal.

The impact of adding different concentrations of IAA on the activity of major fiber-degrading enzymes *in vitro* fermentation fluid is shown in Figure 6. A total of 15 and 150 mg/kg IAA significantly increased the activity of xylanase (*p* < 0.05) and showed an enhancing trend for pectinase (*P_L_* = 0.002) and carboxymethyl cellulase (*P_Q_* = 0.005). In contrast, 1,500 mg/kg concentrations of IAA significantly decreased the activity of pectinase (*p* < 0.05), exhibited a certain inhibitory effect on the activity of xylanase and carboxymethyl cellulase.

**Figure 7:**
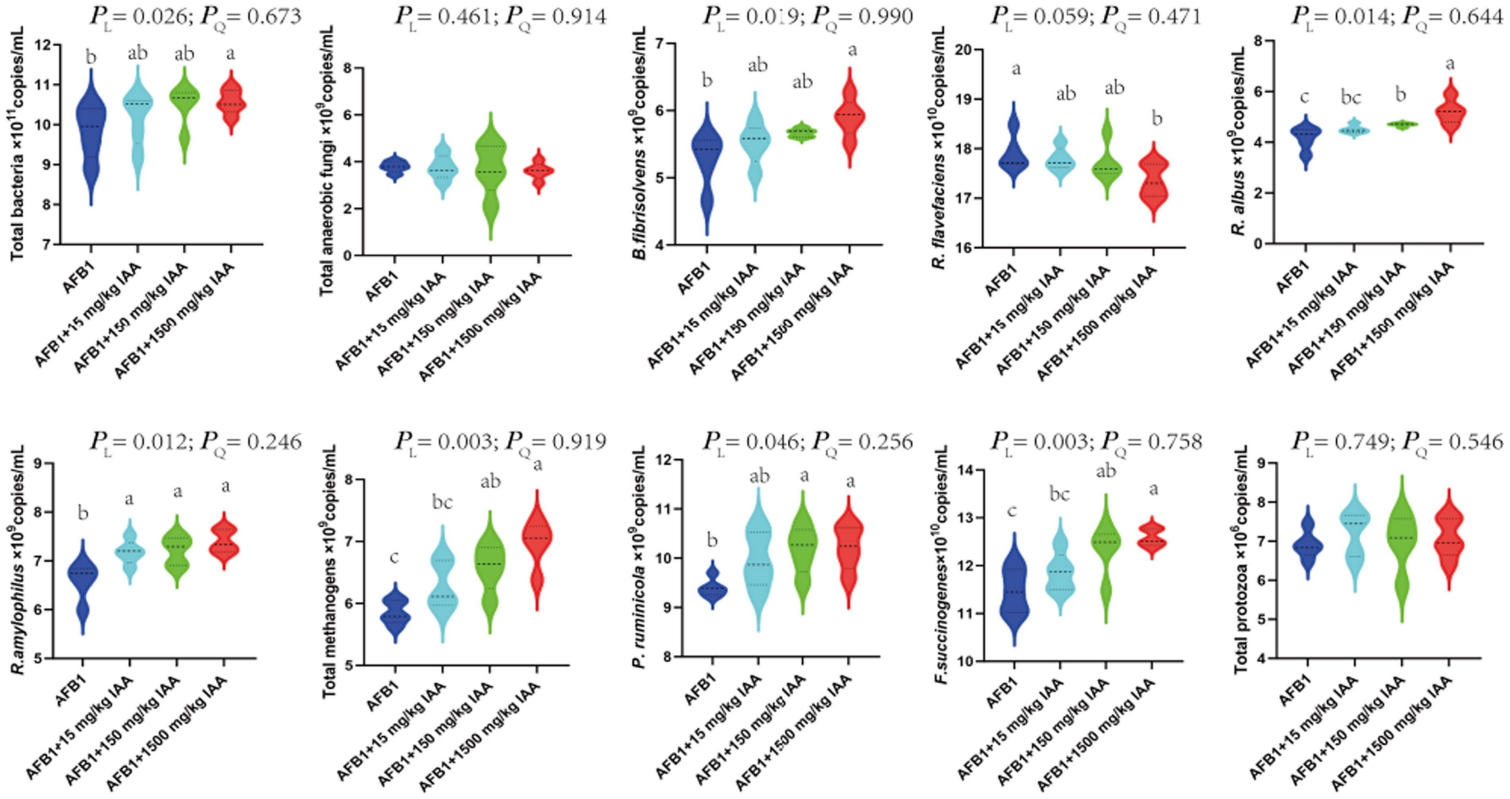
Effect of IAA supplementation on rumen fermentation and AFB1 removal.

That 15, 150 and 1,500 mg/kg of IAA were added to the fermentation fluid, there was an increasing trend in the relative proportions of total bacteria (*P_L_* = 0.026), *B. fibrisolvens* (*P_L_* = 0.019), *R. albus* (*P_L_* = 0.014), *R. amylophilsus* (*P_L_* = 0.012), total methanogenic archaea (*P_L_* = 0.003), *P. ruminicola* (*P_L_* = 0.046) and *F. succinogenes* (*P_L_* = 0.003) (Figure 7). However, the relative proportions of *R. flavefaciens* had a downward trend (*P_L_* = 0.059).

## Discussion

Previous studies had found that some ruminal microbiota have the ability to removal AFB1 (26, 29). A total 1 mg/kg of AFB1 can cause a disturbance in the rumen microbiota and reduce the abundances of *Prevotella* and *P. butyrivibrio* (30). But higher concentrations (2.5 mg/kg) of AFB1 not only alter rumen fermentation pattern but also compromise the safety of liver function (26). In our study, as the fermentation time increased, AFB1 exposure gradually decreased, reaching a stable state after a period of time. Especially when the fermentation time reached 48 h, the removal efficiency of AFB1 can reach about 80%. This phenomenon can be attributed to two key factors: (1) ruminal microbiota reduce the toxicity of AFB1 through adsorption; (2) enzymes secreted by ruminal microbiota can break down the structure of AFB1, converting it into weakly toxic substances. In addition, research has shown that AFB1, as a harmful secondary metabolite widely present in feed and its raw materials. AFB1 can disrupt the balance of microbiota in the rumen when ingested by ruminants and enter the rumen, leading to change the rumen microbiota and affecting the normal fermentation process of the rumen (31, 32). In our study, the pH showed no significant changes, indicating that the *in vitro* fermentation test was normal. VFAs are the main source of energy for ruminants to obtain from feed (33), but the exposure of AFB1 has a significant inhibitory effect on the rumen microbiota, leading to decrease in the total volatile fatty acids (TVFA) and change in the fermentation type. This corresponds to previous studies by Cao et al. (30). *In vitro* fermentation experiments showed that after AFB1 exposure, the relative proportions of core microbiota in the rumen such as *Prevotella* and *Fusobacterium* decreased, these presented the similar results as acetic acid changes. In addition, AFB1 also reduced the activity of xylanase, consistent with the changes of *B. fibrisolvens*, while the activity of other enzymes such as pectinase did not change significantly, which may be related to the amount of AFB1 exposure. AFB1 exposure can lead to produce changes in pectase activity (34). Although the removal mechanism of AFB1 unclear, growing evidence has revealed that rumen microbiota dysbiosis is involved in the exposure of AFB1. Therefore, the restoration of rumen microbiota balance is likely to contribute to AFB1 removal.

IAA is a crucial biological regulatory substance for the growth of plants, which is considered to be an effective and environmentally friendly additive with the potential to modulate the balance of animal microbiota. Although research on IAA in rumen microbiota is still in its infancy, this experiment has confirmed that adding different concentrations of IAA to rumen fluid containing AFB1 can effectively reduce the content of AFB1. IAA can activates the expression of cytochrome genes, such as cytochrome P450 enzymes have been found in different organisms, that is a key enzyme that involve in the biotransformation of AFB1 and affects its toxicity (17). Moreover, IAA can maintain microbiota homeostasis and improve microbiota disorders by activating signal transduction pathways through its specific ligands (35). Although the mechanism by which IAA removes AFB1 is not yet clear, a growing body of evidence suggests that the rumen microbiota is involved in the removal of AFB1. Therefore, we have focused on the metabolic mechanisms of rumen microbiota with the addition of IAA. Considering that high concentrations of IAA may affect the balance of the rumen, according to the “Safety Use Specifications for Feed Additives” the recommended amount of tryptophan (the precursor of IAA) in the diet of ruminants is 0.1% (1 g/kg). The dosage of IAA is based on previous reports (50 mg/kg body weight) (36) and our preliminary studies, and with a rumen volume of 50 L, it is considered safe for cattle. In addition, some studies have shown that excessively high concentrations (50 mg/kg body weight) of IAA may change the animal microbiota (19). Therefore, this experiment further explored the specific impact of IAA additions of 15, 150, and 1,500 mg/kg on rumen fermentation parameters, aimed to verify the addition of a reasonable range of IAA concentrations to mitigate the impact of AFB1 on the rumen fermentation process, thereby enhancing animal health.

In our study, found that 150 and 15 mg/kg concentrations of IAA can significantly increase the content of TVFA, while 1,500 mg/kg concentrations of IAA tend to decrease. Indicate that the addition of IAA, enhances the rumen microbiota ability to utilize carbon sources and stimulates the degradation of nutrients within the rumen (37). In the experiment, after the addition of IAA, the content of acetic acid showed a linear upward trend, this is attributed to the metabolic fermentation products of *R. amylophilus* and *P. ruminicola* being primarily acetic acid (38, 39). With the addition of IAA, the observed changes in the microbiota, such as *Prevotella* and *Fusobacterium*, are closely related to the adjustment of VFAs composition (40, 41). These microbiota decompose the fiber in the feed, promoting the production of acetic acid and propionic acid. Propionic acid is key in the gluconeogenesis process, affecting body fat and lactose synthesis, this is primarily due to *F. succinogene* impact on the production of propionic acid through the propionate metabolism pathway (42–46). The concentration of IAA has a significant regulatory effect on propionic acid production, with 150 and 15 mg/kg concentrations of IAA causing a linear increase in propionic acid content, while 1,500 mg/kg concentrations of IAA reduce the level of propionic acid. *F. succinogene* also exhibited a trend of increasing first and then decreasing in this study. The change in butyric acid content is also related to the amount of IAA added, with 150 mg/kg and 15 mg/kg concentrations of IAA increasing butyric acid content, while 1,500 mg/kg concentrations of IAA reduce butyric acid content. *B. fibrisolvens* metabolize the production of butyric acid in rumen fluid, and the addition of 1,500 mg/kg IAA may make the microbiota rapidly metabolize and compete for rumen nutrients, resulting in the poor competitiveness of *B. fibrisolvens* and reducing the production of butyric acid (47). In addition, the regulation of the acetic acid/propionic acid ratio affects microbiota protein synthesis and the structure of the rumen microbiota, which relates to the digestion and nutritional metabolism of the whole body (48, 49). In the rumen ecosystem, bacteria can degrade and utilize starch and plant cell wall polysaccharides, such as xylan and pectin. These bacteria play an important role in the degradation of protein and the absorption and fermentation of peptides (50–53). The addition of IAA has a significant impact on the changes in the rumen microbiota and the activity of the main fiber-degrading enzymes. A total of 15 and 150 mg/kg concentrations of IAA had increased trend of the activity of pectinase, xylanase, and carboxymethyl cellulase, while 1,500 mg/kg concentrations of IAA reduced the activity of these enzymes. These results show that IAA alleviates the imbalance of the rumen fermentation caused by AFB1 by regulating the rumen microbiota and enzyme activity. *R. albus*, *B. fibrisolvens* and *R. flavefaciens*, as the main fiber-degrading bacteria, can produce xylanase. In this study, the significant increase in *R. albus* and *B. fibrisolvens* is consistent with the changes in xylanase. The decrease in xylanase with the addition of 1,500 mg/kg concentrations of IAA may be related to the downward trend of *R. flavefaciens*. The changes in carboxymethyl cellulose are mainly caused by *P. ruminicola*, and pectinase follows the relative changes of *R. flavefaciens* and *B. fibrisolvens* (36). In summary, the appropriate addition of IAA had a positive impact on the rumen microbiota and metabolic products, but 1,500 mg/kg concentrations of IAA may inhibit the rumen fermentation process. These findings provide important information for optimizing rumen fermentation and improving the nutritional absorption of ruminants.

## Conclusion

This experiment preliminarily explored the impact of *in vitro* addition of IAA on the removal rate of AFB1 and rumen fermentation. It was found that IAA in the range of 150 mg/kg addition could improve the removal rate of AFB1 and reduce the negative effects of AFB1 on *in vitro* fermentation characteristics and fermentation end-products. This provides a new strategy to mitigate the potential threat of AFB1 to animal health.

## Data Availability

The original contributions presented in the study are included in the article/supplementary material, further inquiries can be directed to the corresponding authors.
